# Angioleiomyoma: How to Suggest the Diagnosis on Imaging?

**DOI:** 10.5334/jbsr.4116

**Published:** 2025-10-28

**Authors:** Elena Enriquez, Filip M. Vanhoenacker

**Affiliations:** 1 University of Antwerp, Faculty of Medicine and Health Sciences, 2000 Antwerpen, Belgium; 2 AZ Sint-Maarten Mechelen and University (Hospital) Antwerp and Ghent, Liersesteenweg 435, 2800 Mechelen, Belgium

**Keywords:** angioleiomyoma, US, MRI

## Abstract

*Teaching point:* An angioleiomyoma is a benign superficial tumor that presents with relatively characteristic features on US and MRI.

## Case

A 40-year-old man presented with a slow-growing soft tissue lump on the palmar side of the left wrist accompanied by cold-induced pain for over 6 years.

**Figure 1 A–C.**
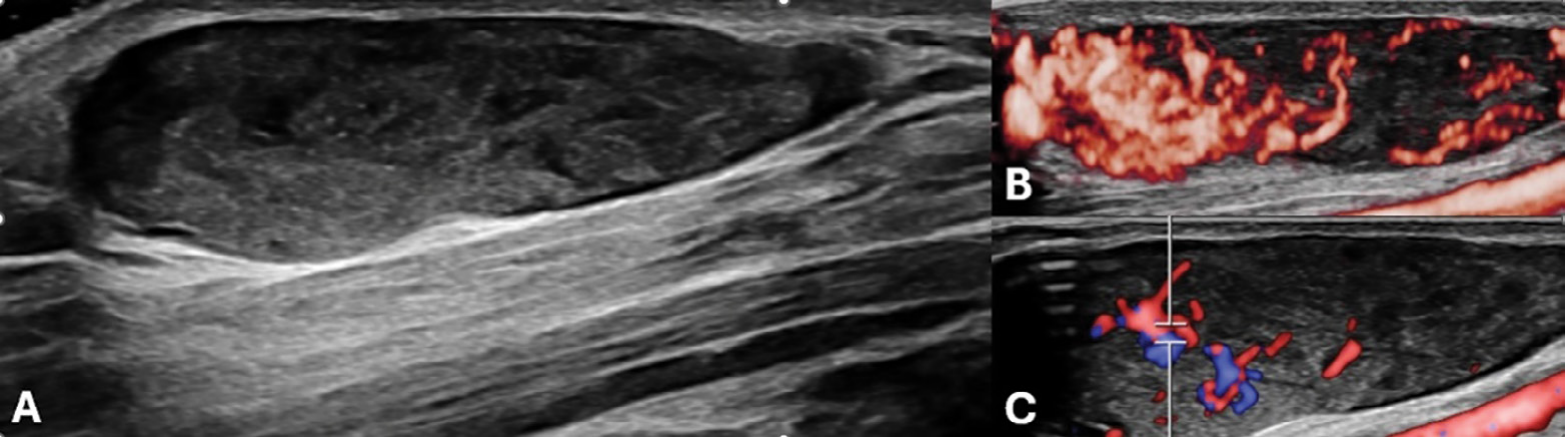
Doppler-Ultrasound: hypoechogenic lesion with tiny intralesional anechoic foci and extensive vascularization.

Grey-scale ultrasound (US) demonstrated an oval hypoechogenic lesion with tiny intralesional anechoic foci superficial to the flexor fascia (Figure 1A). Subsequent power Doppler and color Doppler showed extensive vascularization (Figure 1B) with arterial and venous signals (Figure 1C).

**Figure 2 A–B.**
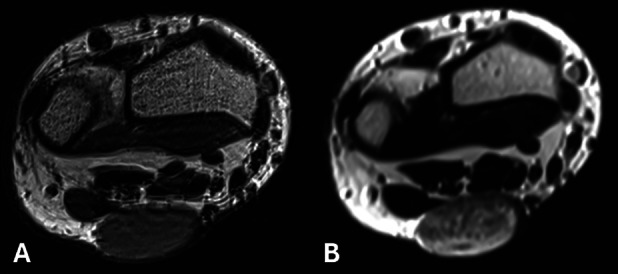
MRI. The lesion is slightly hyperintense compared to muscle on T1-WI and heterogeneous on T2-WI.

**Figure 3 A–B.**
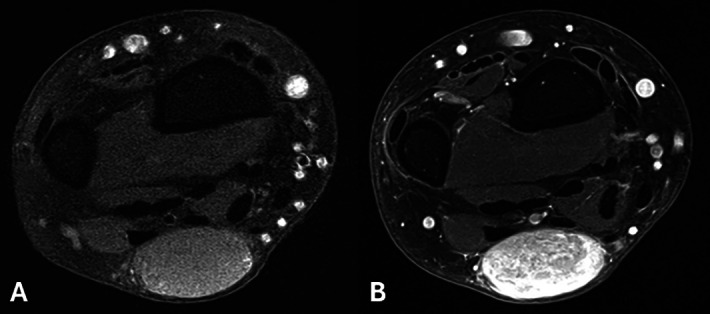
MRI. T1-WI before and after gadolinium contrast shows marked enhancement.

Magnetic resonance imaging (MRI) revealed a well-defined lesion, appearing slightly hyperintense compared to muscle on T1-weighted images (WI) (Figure 2A) and slightly heterogeneous hyperintense on T2-WI with multiple hypointense foci (Figure 2B) and a subtle incomplete peripheral rim of low signal. After intravenous administration of gadolinium contrast, marked enhancement of the lesion was seen (Figure 3A-B).

Since malignancy could not be definitively ruled out, a biopsy was performed. The diagnosis of angioleiomyoma was made.

## Comment

An angioleiomyoma is a benign tumor arising in the subcutis or dermis almost always located at the extremities [1]. It originates from the smooth muscles of blood vessels and can be categorized into three subtypes (solid, venous and cavernous), depending on the predominant type of vessel involved. The lesion mostly presents in the fourth to sixth decades of life. An angioleiomyoma grows slowly and may be painful [1].

The etiology of an angioleiomyoma is unknown. The differential diagnosis includes neural sheath tumors, hemangiomas, inclusion epidermoid cysts, tenosynovial giant cell tumors, among others [1].

Conventional radiographic and computed tomographic (CT) scans are nonspecific. Intralesional calcifications are rare [1].

Ultrasound shows a superficial, well-defined, predominantly hypoechogenic lesion with tiny anechoic foci, which correspond to vessels [1]. The lesion is hypervascular on Doppler with mixed arterial and venous signals. Sometimes a feeding vessel can be visualized [1].

MRI likewise demonstrates a superficial and well-defined lesion, which is typically isointense or mildly hyperintense compared to skeletal muscle on T1-WI. On T2-WI, an angioleiomyoma is of intermediate to high signal with intralesional hypointense foci, which may result in a dark reticular appearance [1]. These foci are related to thrombosed small vascular structures or fibrous tissue. A peripheral dark rim may be seen on both pulse sequences. Vivid homogeneous or heterogeneous enhancement is seen [1].

The combination of a slow-growing superficially located and well-defined mass at the extremities, the presence of a dark reticular sign and peripheral dark rim on T2-WI and the hypervascular nature may be useful to suggest an angioleiomyoma in the differential diagnosis [1].

However, as imaging features are not pathognomonic, histological examination is recommended. Surgical excision is the treatment of choice.
